# Identification of an anoikis-related gene signature and characterization of immune infiltration in skin cutaneous melanoma

**DOI:** 10.1097/MD.0000000000037900

**Published:** 2024-04-26

**Authors:** Ziqian Xu, Juntao Huang, Weimin Shi, Ying Qi, Feng Yuan, Bingjiang Lin

**Affiliations:** a Department of Dermatology, The First Affiliated Hospital of Ningbo University, Ningbo, China; b Department of Otolaryngology Head and Neck Surgery, Ningbo Medical Center Lihuili Hospital, The Affiliated Lihuili Hospital of Ningbo University, Ningbo, China; c Department of Dermatology, Shanghai General Hospital, Shanghai Jiao Tong University School of Medicine, Shanghai, China.

**Keywords:** anoikis, immunotherapy, prognosis, risk score, skin cutaneous melanoma

## Abstract

Anoikis is considered strongly associated with a biological procession of tumors. Herein, we utilized anoikis-related genes (ARGs) to predict the prognosis and immunotherapeutic efficacy for skin cutaneous melanoma (SKCM). RNA-seq data were obtained from The Cancer Genome Atlas and Gene Expression Omnibus databases. After dividing patients into novel subtypes based on the expression of prognostic ARGs, K–M survival was conducted to compare the survival status. Subsequently, differentially expressed ARGs were identified and the predictive model was established. The predictive effects were validated using the areas under the curve about the receiver operating characteristic. Moreover, tumor mutation burden, the enriched functional pathway, immune cells and functions, and the immunotherapeutic response were also analyzed and compared. The distribution of model genes at cell level was visualized by the single-cell seq with tumor immune single-cell hub database. Patients of The Cancer Genome Atlas–SKCM cohort were divided into 2 clusters, the cluster 1 performed a better prognosis. Cluster 2 was more enriched in metabolism-related pathways whereas cluster 1 was more associated with immune pathways. A predictive risk model was established with 6 ARGs, showing the areas under the curves of 1-year, 3-year, and 5-year ROC were 0.715, 0,720, and 0.731, respectively. Moreover, risk score was negatively associated with tumor mutation burden and immune-related pathways enrichment. In addition, patients with high-risk scores performed immunosuppressive status but the decreasing scores enhanced immune cell infiltration, immune function activation, and immunotherapeutic response. In this study, we established a novel signature in predicting prognosis and immunotherapy. It can be considered reliable to formulate the complex treatment for SKCM patients.

## 1. Introduction

Skin cutaneous melanoma (SKCM) is a type of highly malignant skin tumor originating from melanocytes, and its mortality can be as high as 75%.^[1]^ At present, SKCM has become the third most common tumor in the United States. In 2019, there were approximately 192,000 new cases, and its overall incidence has increased by approximately 6 times compared with 40 years ago, and the number of new cases around the world is growing.^[2]^ Early-stage and localized SKCM displays a favorable clinical outcome with surgical resection, and the 5-year survival rate can be as high as 99%. Nevertheless, SKCM is prone to distant metastasis, once it occurs, the survival rate drops to only 15%.^[3]^ Melanoma is prone to metastasize, once diagnosed, tumor cells have already spread to lymph nodes, adjacent tissues, or distant organs.^[4]^ In the past few decades, some immunotherapies aimed at metastatic melanoma have been developed and have achieved good curative effects,^[5]^ among which the most effective one is immune checkpoint inhibitors (ICIs). ICIs aim to reactivate the body’s antitumor immune response by blocking co-inhibitory signals.^[6,7]^ Given the shortcomings of drug resistance and unresponsive treatment with clinical classical ICIs in a considerable number of advanced SKCM patients,^[8,9]^ uncovering the underlying mechanism of tumor metastasis and establishing a stable multibiomarker signature to forecast the prognosis of SKCM patients are extremely necessary.

Cells in normal tissue utilize very special extracellular matrix (ECM) attachments, and the incorrect types of ECM may lead to the same consequence as without ECM. Anoikis is a newly programmed cell death (PCD) that occurs when cells detach from the ECM, thus preventing cells epithelial cells from colonizing elsewhere.^[10]^ The displaced cells can be effectively removed through anoikis, thus preventing them from reattaching to the new matrix and their maldevelopment. Anoikis is an emerging hallmark in health and diseases.^[11]^ Cancer cells are not sensitive to ECM, so they can survive and proliferate without adhesion to ECM, which makes anoikis resistance a natural molecular prerequisite of the invasiveness and diffusivity of cancer.^[12]^

Anoikis was considered to be regulated via both intrinsic and extrinsic pathways. In the intrinsic pathway, the Bcl-2 protein family controls the formation of pores in the outer mitochondrial membrane (OMM), and then releases cytochrome c and increases mitochondrial permeabilization.^[13]^ The exogenous pathway starts by connecting death receptors (such as TNFR or Fas receptor) on the cell surface, leading to the formation of a death-inducing signaling complex (DISC).^[14]^ Both these pathways can activate caspase and downstream signaling pathways, finally initiating the loss-of-nest apoptosis program.^[15]^

In previous studies, anoikis has been shown to be associated with the various tumor metastasis, such as gastric cancer,^[16]^ lung cancer,^[17]^ and colon cancer.^[18]^ For melanoma, several studies have also been devoted to exploring the relevant signal pathways that regulate apoptosis resistance-related metastasis of melanoma. Melanoma requires B-RAF and PI-3 kinase signaling for protecting it from anoikis.^[19]^ Imp1 interacts with beta-1 integrin and CD63 confers anoikis resistance to melanoma by activating the PI3-K signaling pathway.^[20]^ Anoikis is critical for resistance to tumor progression and metastatic spreading and is essential for tissue homeostasis and body development.^[12]^ At present, studies have proved that there was some certain relationship between the highly metastatic characteristics of melanoma with anoikis, but whether anoikis-related genes (ARGs) can be used as prognostic indicators of SKCM is rarely investigated.

In our study, we conducted a systematic and comprehensive analysis of transcript and clinical data from The Cancer Genome Atlas (TCGA) database, and constructed a predictive model consisting of ARGs. The model was found to be a reliable independent prognostic factor for SKCM. A nomogram comprehensively evaluated the predictive ability of risk model scores and other clinical factors for the prognosis of SKCM. In addition, we also explored the difference in tumor mutation, tumor immune microenvironment (TME), immunotherapy response between high-risk and low-risk groups, which was of certain significance for guiding the diagnosis and treatment of SKCM.

## 2. Methods and materials

### 2.1. Obtainment of the SKCM dataset and anoikis-related gene (ARG) set

The RNA-seq data for the SKCM cohort were downloaded from TCGA database (last assessed: September 1, 2022) in fragments per kilobase million format, consisting of 472 SKCM samples and 1 normal sample. Clinical details about TCGA–SKCM cohort, including survival status, overall survival (OS) time, age, sex, TMN stage, and clinical stage. In addition, the information about progression-free survival (PFS) and disease-specific survival (DSS) was also obtained from the TCGA Pan-Cancer dataset. In addition, data about patients in GSE65904 cohort from the Gene Expression Omnibus (GEO) were extracted as an external test dataset.

Subsequently, we searched the ARGs by screening the GeneCards database with the keywords anoikis, and those with a relevance score > 0.4 were identified as the eligible ARGs for further analysis by referring to the previous studies.

### 2.2. Construction of SKCM subtypes based on the expression of prognostic ARGs

With the criteria of *P* value < .01, the survival-related ARGs were selected as prognostic ARGs based on univariate Cox (uni-Cox) proportional hazard regression analysis via the “limma” and “survival” R packages. Subsequently, according to the expression of these prognostic ARGs, nonnegative matrix factorization (NMF) analysis was applied to divide patients into 2 subtypes with the use of the “NMF” R package. Kaplan–Meier (K–M) survival analysis were constructed to compare the differences of OS, PFS, and DSS between the 2 subtypes. Moreover, gene set variation analysis (GSVA) was conducted to explore differential KEGG enrichment pathways in clusters.

### 2.3. Establishment of a predictive signature and correlation analysis with clinicopathological features

With the criteria of |log2 fold change (logFC)| > 0.585 and false decrease rate (FDR) < 0.05, the differentially expressed ARGs (DEARGs) were selected, and the protein–protein interaction network was analyzed by STRING online database and visualized by Cytoscape version 3.7.2 software. Based on the results of uni-Cox hazard analysis (*P* < .01), the prognostic DEARGs were then selected by the least absolute shrinkage and selection operator (LASSO) analysis with 10-fold cross-validation. Multivariate Cox regression analysis was applied to further identify eligible DEARGs and calculate the coefficient index of the novel risk model. Patients were then assessed by the above risk model with the following formula: Anoikis Risk Score (ARS) = Σ coef (genei) × exp (genei), and they are regrouped into the low-ARS and high-ARS groups based on the median of ARS of the cohort. Referring to the K–M survival analysis, the survival comparison between the 2 risk groups was investigated to explore the differences of the OS, PFS, and DSS. In addition, the survival receiver operating characteristic (ROC) curves were applied to assess the 1-year, 3-year, and 5-year predictive effects of the TCGA–SKCM and GSE65904 cohorts concerning on the value of their areas under the curves (AUCs). The C-index was used to compare the predictive effects of the ARS system to other clinical characteristics. Similarly, the comparison of predictive differences among the risk model and other clinicopathological features were also compared by the value of AUCs, uni-Cox, and multi-Cox analysis for further assessment. The independent predictive factors (*P* values were <.05 in both uni-Cox and multi-Cox analysis) were then pooled to establish a nomogram for prediction.

Furthermore, the distinguishment of ARS in different clinicopathological characteristics was analyzed by the Wilcoxon test, and the differences of OS in various clinical subgroups were compared via the log-rank test and K–M survival analysis.

### 2.4. Functional enrichment analysis and tumor burden mutation (TMB) comparison

GSVA was also performed to compare the differentially enriched pathways (adjusted *P* value < .05) between the low-ARS and high-ARS groups.

Additionally, the mutation rate of the model ARGs in SKCM was calculated. To compare the differences of TMB between the 2 risk groups, the 20 topmost mutated genes of TCGA–SKCM in both the low-ARS and high-ARS groups are shown in the waterfall plots. Additionally, the correlation between ARS and TMB was analyzed based on the Spearman correlation test, and the survival analysis was performed for patients based on ARS combined with TMB.

### 2.5. Exploration of the immune infiltrated landscape

To further explore the TME in the risk model, the gene expression of model ARGs in immune cells around the tumor sample was assessed with the use of 2 single-cell RNA sequencing datasets (GSE115978 and GSE120575) via the tumor immune single-cell hub (TISCH, http://tisch.comp-genomics.org/home/) database.

Similarly, the differences in immune cell infiltrated status between the low-ARS and high-ARS groups were assessed using the CIBERSORT platform and compared via the Wilcoxon test, and the correlation rate between ARS and immune cells was analyzed by Spearman’s test. In addition, the differences in immune functions between the 2 risk groups were assessed via the single sample gene set enrichment analysis (ssGSEA) algorithm and the TME scores (including immune scores, stromal scores, ESTIMATE scores, and tumor scores) were calculated with the application of the “estimate” R package.

### 2.6. Assessment of predictive effects on immunochemotherapeutic response

To further assess the predictive effects of the risk model on the ICI-therapeutic results, we calculated and compared the expression levels of immune checkpoint-related genes between the low-ARS and high-ARS groups via the Wilcoxon test. After respectively dividing 2 immunotherapy cohort (GSE78220 and MSKCC-SKCM) into low-risk and high-risk groups concerning the novel ARS systems, we explored and compared the distinguish of immunotherapy results (response or non-response) between the risk groups. Moreover, to further evaluate the ARS model on predicting the immunotherapeutic response to PD-1 and CTLA-4 inhibitor therapy, the immunophenoscore (IPS) of each SKCM patient was analyzed by the TCIA database (https://tcia.at/home) and compared between the 2 ARS groups.

## 3. Results

### 3.1. Obtaining the ARG set and molecular subtypes

**Figure 1.**
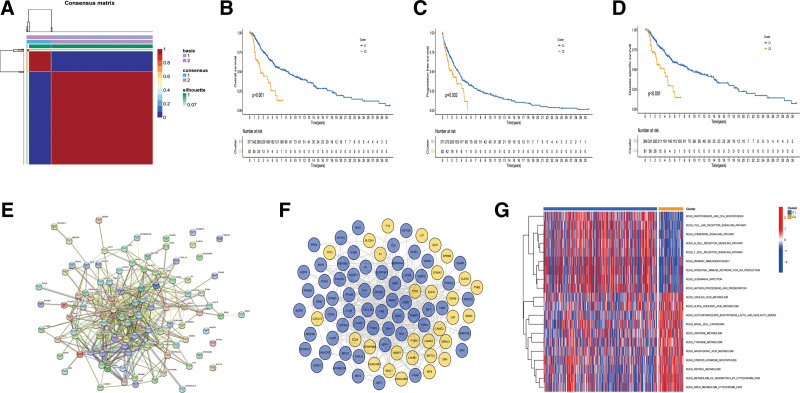
Constructing tumor subtypes based on expression of prognostic anoikis-related genes (ARGs). (A) Clusters heatmap based on nonnegative matrix factorization; (B) comparison of overall survival in clusters; (C) comparison of progression-free survival in clusters; (D) comparison of disease-specific survival in clusters; (E) protein–protein interaction network of differentially expressed ARGs in clusters; (F) visualization of network about differentially expressed ARGs in clusters, the blue cycles were upregulated in cluster 1 whereas yellow cycles were downregulated; (G) gene set variation analysis about pathways between clusters 1 and 2.

Concerning the relevance ratio > 0.4, a total of 496 ARGs were selected by screening the GeneCards database (Table S1, Supplemental Digital Content, http://links.lww.com/MD/M224). Subsequently, with the criteria of a *P* value < .01, 79 ARGs were identified as survival-related genes, and patients in the TCGA–SKCM dataset were regrouped into 2 different clusters based on the expression of these ARGs (Fig. 1A). The K–M survival analysis suggested that patients in cluster 1 exhibited better prognosis than those in cluster 2 (Fig. 1B). Similarly, while comparing the difference in PFS and DSS between the 2 clusters, patients in cluster 1 also had a better survival status than those in cluster 2 (Fig. 1C and D). Moreover, the protein–protein interaction network of DEARGs was analyzed by the STRING database and revisualized by Cytoscape version 3.6.2 software, showing blue cycles upregulated in cluster 1, whereas yellow cycles downregulated (Fig. 1E and F). In addition, as indicated by the GSVA, cluster 1 was much more enriched in immune-related pathways (e.g., the B-cell receptor signaling pathway and T-cell receptor signaling pathway); however, cluster 2 showed more activation of metabolism (e.g., histidine and tyrosine metabolism) (Fig. 1G).

### 3.2. Establishment and verification of the predictive risk model

**Figure 2.**
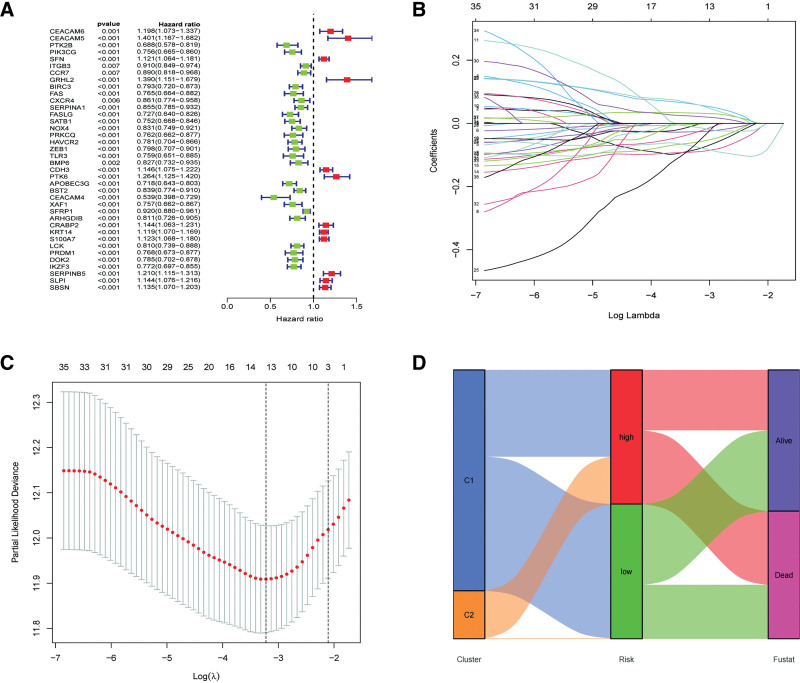
Establishing a predictive risk model according to differentially expressed ARGs. (A) Forest plot of survival-related AGRs in TCGA–SKCM cohort; (B) LASSO diagram for ARGs; (C) cross-validation curve for prognostic ARGs; (D) Sankey diagram to reflect the relationship between the clusters, risk groups, and survival status. ARGs = anoikis-related genes, LASSO = least absolute shrinkage and selection operator, SKCM = skin cutaneous melanoma, TCGA = The Cancer Genome Atlas.

**Figure 3.**
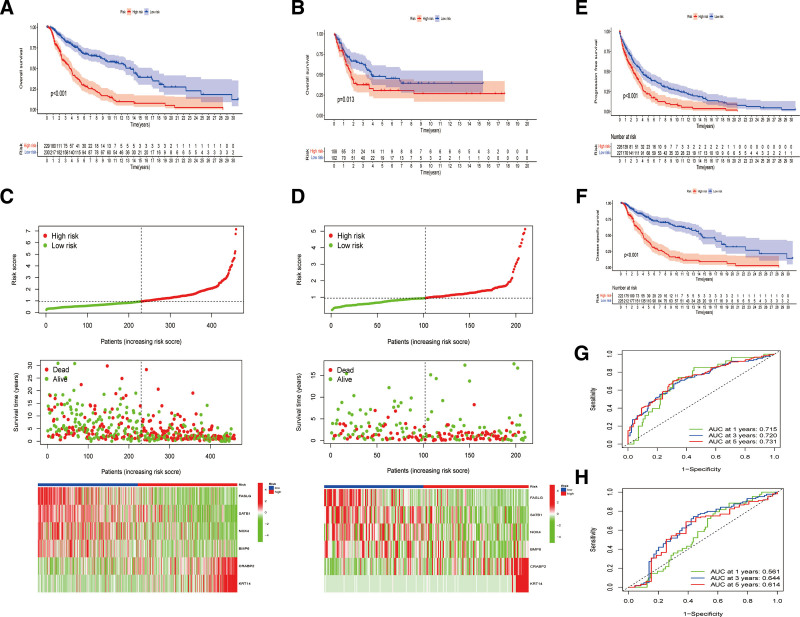
Validation of risk model. (A) K–M survival comparison of overall survival (OS) in risk groups in TCGA–SKCM cohort; (B) comparison of OS in GSE65904 cohort; (C) exhibition of risk scores, survival time and status, and distributing heatmap of model genes in TCGA–SKCM cohort; (D) exhibition of risk scores, survival time and status, and distributing heatmap of model genes in GSE65904 cohort; (E) K–M survival curve about progression-free survival in ARS groups; (F) comparison of disease-specific survival in risk groups. (G) ROC curves for 1-year, 3-year, and 5-year survival in TCGA–SKCM cohort; (H) ROC curves for 1-year, 3-year, and 5-year survival in GSE65904 cohort. ARS = Anoikis Risk Score, ROC = receiver operating characteristic, SKCM = skin cutaneous melanoma, TCGA = The Cancer Genome Atlas.

The uni-Cox hazard regression analysis was conducted to select eligible DEARGs with a *P* value < .01. As indicated by the forest plot in Figure 2A, 12 ARGs possibly increase the risk of SKCM patients (e.g., CEACAM6); nevertheless, 26 ARGs may prolong overall survival. Given this, LASSO regression analysis was conducted to further select the potential model genes and avoid overfitting (Fig. 2B and C). Based on the above results, 6 AGRs were selected to establish the predictive signature, including FASLG, SATB1, NOX4, BMP6, CRABP2, and KRT14. and their coefficients were calculated using the multi-Cox regression. Patients in the TCGA–SKCM and GSE datasets were then assessed by the following formula: anoikis-related risk score (ARS) = FASLG × (−0.244029277099965) + SATB1 × (−0.159056028262982) + NOX4 × (−0.113416813471962) + BMP6 × (−0.117120549833374) + CRABP2 × 0.109587772592032 + KRT14 × 0.0527843178021753 and were subsequently divided into the low-ARS and high-ARS groups based on the median value of ARSs. As indicated by the Sankey diagram (Fig. 2D), patients in the low-ARS groups nearly consisted of those in cluster 1, which had a longer survival time and better survival status; however, patients in cluster 2 were mostly regrouped into the high-ARS groups, which coincided with the K–M survival results, revealing that patients in the low-ARS group had a better prognosis than those in the high-ARS group in both the training (*P* < .001) and test (*P* = .013) cohorts (Fig. 3A and B). In addition, as shown in Figure 3C and D, patients can be clearly distinguished by the risk model. Moreover, the comparison of PFS and DSS also determined that low ARS was associated with a better prognosis (Fig. 3E and F). The 1-year, 3-year, and 5-year AUCs of the ROC curve for the risk model in the TCGA–SKCM dataset were 0.715, 0,720, and 0.731, respectively, as well as 0.561, 0.644, and 0.614 in the external cohort of GSE65904, indicating satisfactory predictive effects of the risk model (Fig. 3G and H).

### 3.3. Correlation analysis between ARSs and clinicopathological features and construction of the nomogram

**Figure 4.**
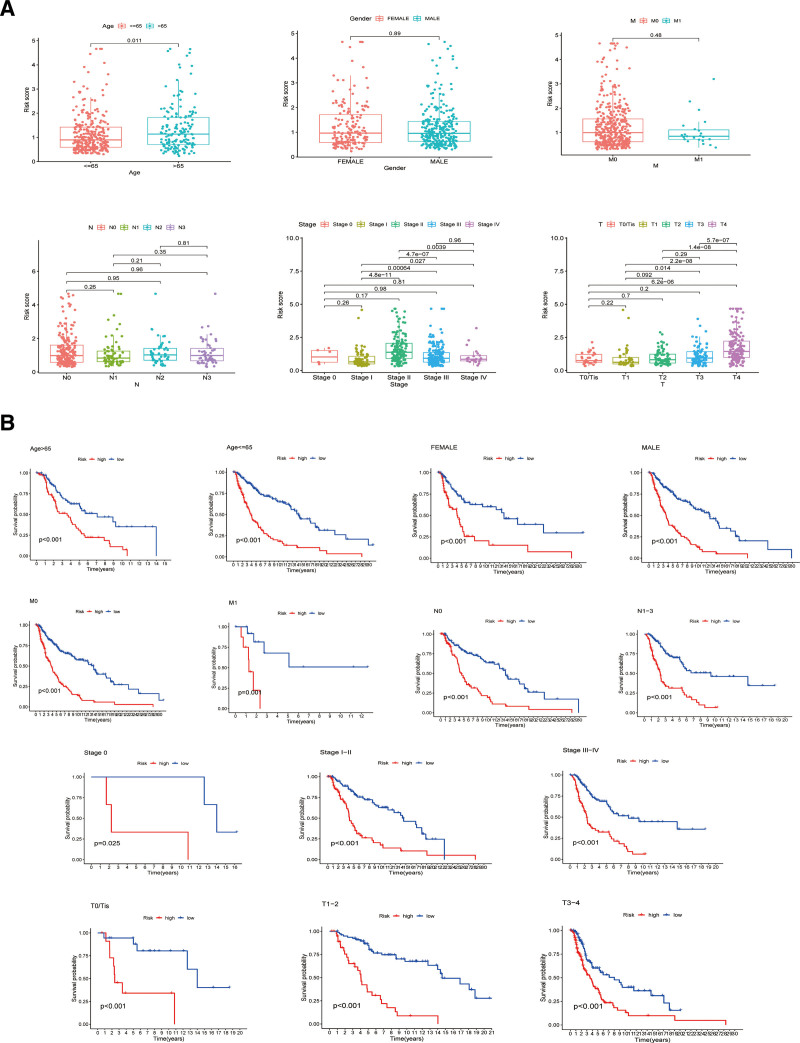
Relationship between ARS and clinicopathological features. (A) Distribution of ARS in different characteristics. (B) K–M survival comparison between the low-ARS and high-ARS groups in different clinicopathological subgroups. ARS = Anoikis Risk Score.

**Figure 5.**
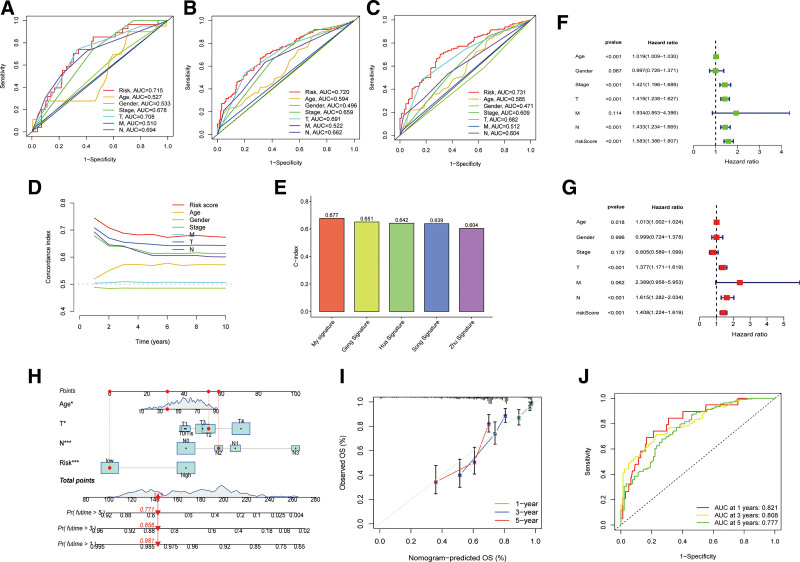
Construction of predictive nomogram. (A) 1-year; (B) 3-year; (C) 5-year ROC curves between ARS and clinicopathological features; (D) comparison of C-index between ARS and other characteristics; (E) bar chart comparing the C-index of ARS with other signatures; (F) forest plot about uni-Cox analysis; (G) forest plot about multi-Cox analysis; (H) nomogram; (I) calibration plot about 1-year, 3-year, and 5-year predictive effects; (J) the ROC curve of the nomogram. ARS = Anoikis Risk Score, ROC = receiver operating characteristic.

To further explore the relationship between ARSs and clinicopathological features, the distribution of ARSs in different characteristics was compared by the Wilcoxon test. Referring to the results shown in Figure 4A, patients with different ages, stages and T stages performed significantly different ARSs, whereas there were no significant differences in ARSs by sex or M and N stages. Additionally, while conducting the K–M survival analysis according to different clinical features, patients in the high-ARS group also had a worse prognosis (Fig. 4B). Besides, the AUC of the ROC curve for the risk model and clinicopathological characteristics suggested that this ARS system exhibited better predictive effects than other features, with the highest AUC values for the 1-year, 3-year, and 5-year ROC (Fig. 5A–C). Similar results were also obtained by C-index analysis (Fig. 5D). In addition, to further assess the predictive effects of our scoring system, we also compare the C-index with other predictive models based on previous studies via the “survcomp” R packages. The results of horizontal comparison show that the C-index of our signature was higher than the other 4 signatures, which indicated our signature had a more reliable predictive ability.^[21–24]^ (Fig. 5E). Furthermore, uni-Cox and multi-Cox analyses were conducted to select the predictive factors. As suggested by the forest plots in Figure 5F and G, age, T stage, and risk score were considered independent parameters with *P* values < .05 in both uni-Cox and multi-Cox regression analyses, which indicated that older patients with worse T stage and higher ARSs may have shorter survival times and worse prognoses. Given these findings, a nomogram was established for further prognostic prediction (Fig. 5H). The nomogram predictions of the calibration plot in terms of 1-year, 3-year, and 5-year survival rates exhibited a high degree of consistency with actual observations, indicating satisfactory predictive effects and reliability of the nomogram (Fig. 5I). The AUC of the 1-year, 3-year, and 5-year ROC curve for the nomogram suggested the nomogram has reliable predictive ability (Fig. 5J).

### 3.4. Relationship between ARS system and TMB

**Figure 6.**
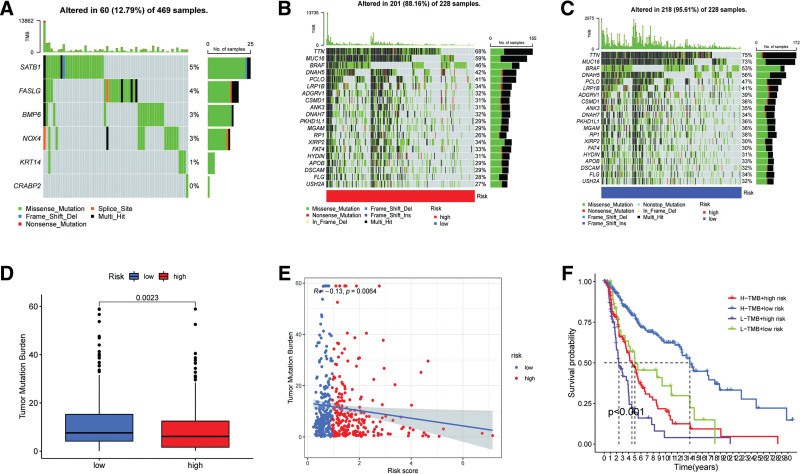
Correlation analysis between ARS and tumor mutation burden (TMB). (A) Mutation frequency of 6 model genes; (B) waterfall plot about frequency of topmost 20 mutated genes in the low-ARS group; (C) waterfall plot about frequency of topmost 20 mutated genes in the high-ARS group; (D) comparison of TMB between the 2 ARS groups; (E) Spearman correlation analysis between ARS and TMB; (E) K–M survival analysis referring to ARS combined TMB. ARS = Anoikis Risk Score.

The mutation frequency of the model gene was assessed and reflected in the waterfall plot, which indicated that the model gene achieved a mutation frequency of 12.79% (60/469) (Fig. 6A). Among them, the SATB1 gene had the highest mutation rate of 5% compared with the other 5 model genes. Similarly, the differences in TMB between the low-ARS and high-ARS groups were assessed, and the top 20 mutated genes in the TCGA–SKCM dataset are also shown in waterfall plots in Figure 6B and C. In addition, as indicated by the Wilcoxon test, patients in the low-ARS group had a higher TMB frequency than those in the high-ARS groups (Fig. 6D). Similar results were also determined by Spearman correlation analysis, which showed that ARS was negatively associated with TMB, showing significant differences (Fig. 6E). Moreover, while combining ARS and TMB to conduct the K–M survival analysis, patients with high ARS and low TMB had the worst prognosis, whereas the low ARS combined with high TMB prolonged the survival time (Fig. 6F).

### 3.5. Functional analysis and tumor immune infiltration landscape

**Figure 7.**
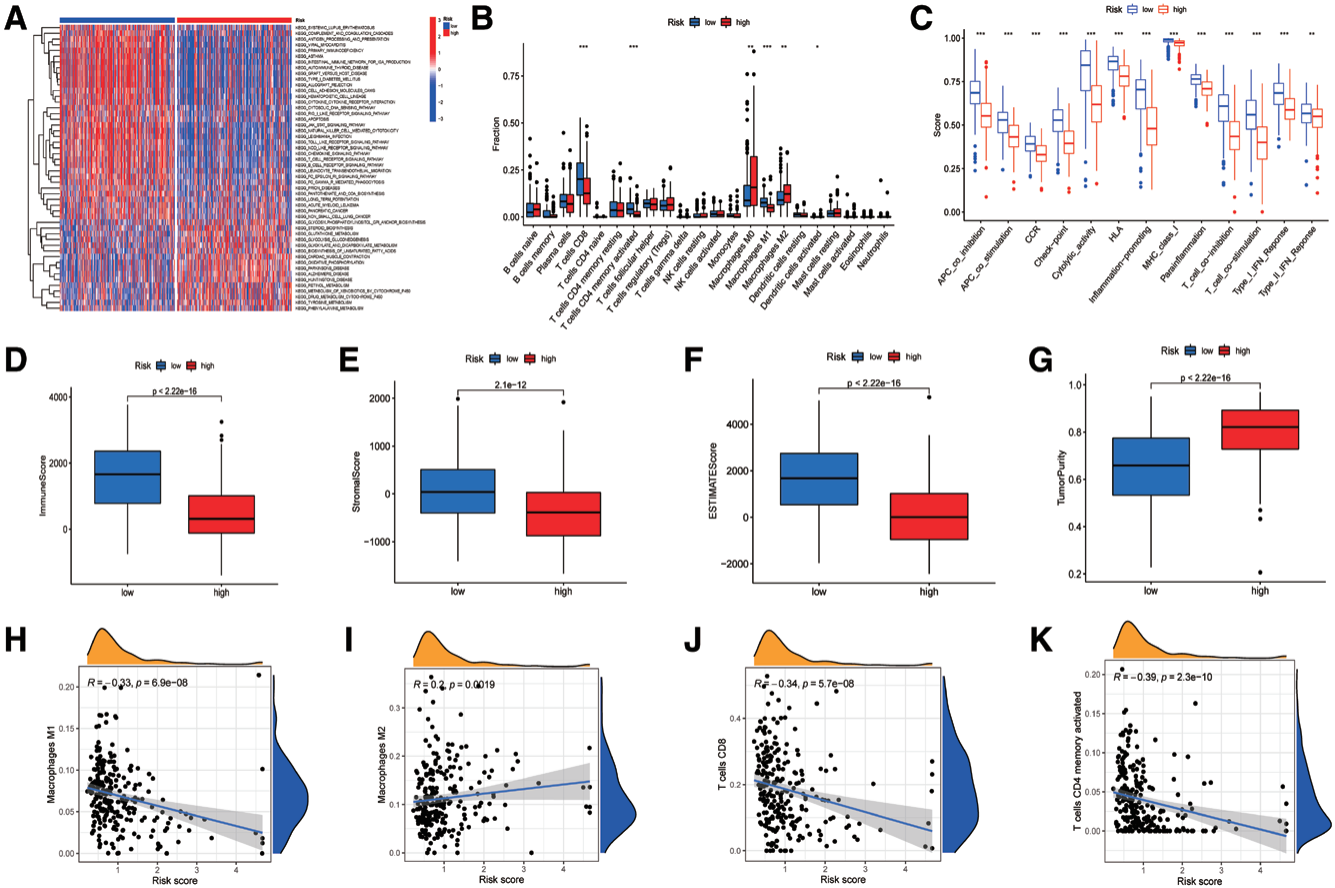
Functional analysis and immune microenvironment. (A) Gene set variation analysis about pathways between the low-ARS and high-ARS groups; (B) immune cell infiltration based on CIBERSORT; (C) comparison of immune function according to ssGSEA analysis; (D–G) correlation analysis between ARS and immune cells, (D) M1 macrophages; (E) M2 macrophages; (F) CD8 T cells; (G) activated memory CD4 T cells; (H–K) TME scores based on estimate platform, (H) immune scores; (I) stromal scores; (J) ESTIMATE scores; (K) tumor purity. ARS = Anoikis Risk Score, ssGSEA = single sample gene set enrichment analysis, TME = tumor immune microenvironment.

According to the GSVA analysis, the enriched KEGG pathways were compared between the low-ARS and high-ARS groups, as shown in the heatmap in Figure 7A. The GSVA results indicated that the low-ARS group had more enriched immune-related pathways, including antigen processing and presentation pathways. Nevertheless, the high-ARS group exhibited more procession associated with metabolism enrichment.

Subsequently, the landscape of immune cell infiltration was assessed with the application of the CIBERSORT platform, suggesting that the low-ARS group had more CD8+ T cells, enriched activated CD4+ memory T cells and enriched M1 macrophages; however, the high-ARS group exhibited more infiltration of M0 and M2 macrophages (Fig. 7B). Besides, based on the results of ssGSEA, patients with low ARSs exhibited more activation of immune functions than those with high ARS (Fig. 7C). Spearman correlation analysis between ARS and immune cell infiltration status concerning the CIBERSORT results also indicated that ARSs were positively correlated with M2 macrophages and negatively associated with M1 macrophages, CD8+ T cells and activated CD4+ memory cells (Fig. 7D–G). Moreover, with the use of the “estimate” R package, patients in the low-ARS group had higher immune scores, stromal scores, and ESTIMATE scores, but the high-ARS group had higher tumor purity (Fig. 7H–K).

### 3.6. Gene expression landscape of cells in the tumor microenvironment

**Figure 8.**
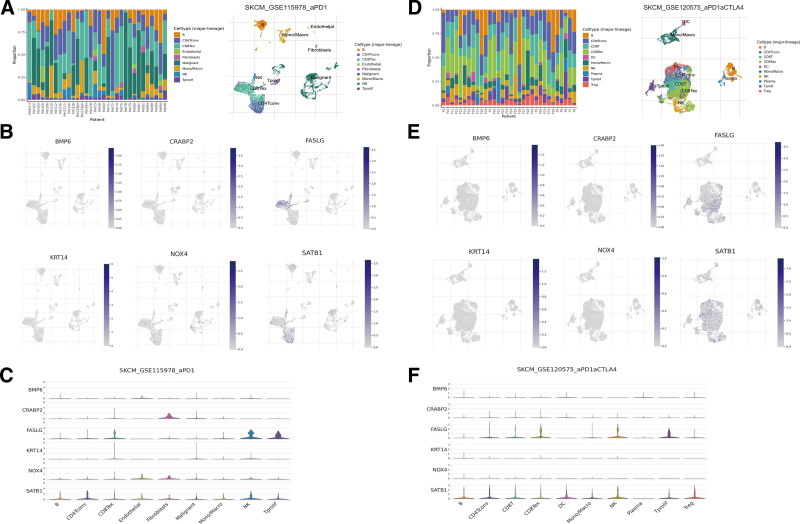
Visualization of 6 model gene expression based on single-cell RNA sequencing datasets. (A) Distributing of cell types in GSE115978 cohort; (B) expression of model genes in different cell types; (C) violin plot about gene expression in cells of GSE115978; (D) distributing of cell types in GSE120575 cohort; (E) expression of model genes in different cell types; (F) violin plot about gene expression in cells of GSE120575.

According to the TISCH database, the expression levels of model genes infiltrating the TME were calculated based on single-cell RNA-seq. Two scRNA-seq datasets (GSE115978 and GSE120675) were assessed, and the cell type and percentage in each sample were calculated, as shown in Figure 8A and D. As indicated by Figure 8B, C, E, and F, NOX4 and CRABP2 were expressed at higher levels in stromal cells (e.g., fibroblasts), whereas FASLAG was expressed at higher levels in immune cells. SATB1 displayed appropriate expression both in immune cells and stromal cells; nevertheless, either BMP6 or KRT14 were expressed at low levels in the TME.

### 3.7. Immunotherapy and chemotherapy

**Figure 9.**
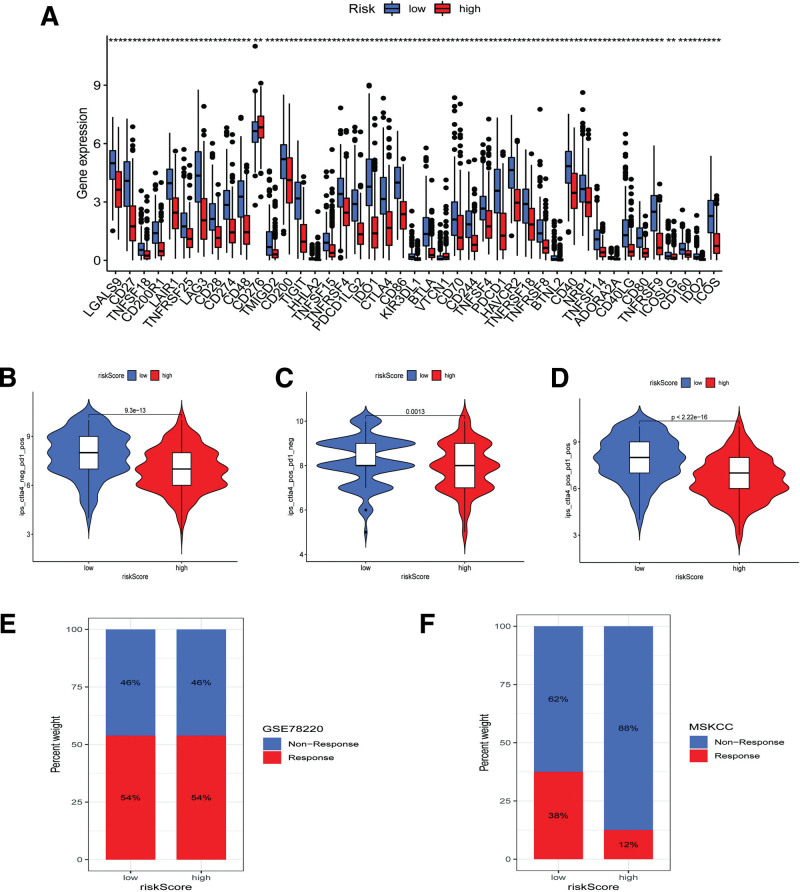
Assessment of immunotherapeutic response. (A) Comparative expression of immune checkpoint inhibitor-related genes in the 2 ARS groups; (B–D) comparison of IPS according to TICA database, (B) PD-1 inhibitor therapy; (C) CTLA-4 inhibitor therapy; (D) treatment combined with PD-1 and CTLA-4 inhibitors, (E) percentage barplot about immunotherapy response in GSE78220 cohort; (F) percentage barplot about immunotherapy response in MSKCC cohort. ARS = Anoikis Risk Score, IPS = immunophenoscore.

According to the results of the Wilcoxon test, the differential expression of immune checkpoint genes was compared between the 2 ARS groups using the “limma” R package (Fig. 9A). Referring to the comparative results, patients in the low-ARS group may have much higher expression of immune checkpoint genes than the high-ARS group, especially CD274 (PD-1), PDCD1, PDCD1LG2, and CTLA-4. Furthermore, to assess the predictive effects of the ARS system in immunotherapy, we used the TCIA database and 2 other external immunotherapy cohorts to compare the potential immunotherapeutic response in the 2 groups. Concerning the results of the TCIA database, patients with a low ARS exhibited a higher IPS whether treated with PD-1 or CTLA-4 inhibitors or combined with them (Fig. 9B–D). However, patients in the GSE78220 cohort exhibited a similar percentage of immunotherapeutic response to PD-1 inhibitor in the 2 ARS groups, but the low-ARS group of the MSKCC cohort displayed a higher percentage of effective response than those with high ARSs. Given the above results, low ARSs may increase the sensitivity of SKCM patients to the immune response (Fig. 9E and F).

## 4. Discussion

Aniokis is a special type of PCD, caused by the incorrect attachment to the ECM or lack of ECM.^[25]^ The pathway was proven to be critical for inhibiting the formation of tumors by neoplastic cells as a powerful protection mechanism of the body.^[11]^ However, the resistance of tumor cells to anoikis through TrkB may lead to tumor metastasis in long-range organs.^[26]^ Owing to its key role in tumor metastasis, anoikis is regarded as a significant step in cancer regulation. At present, most studies mainly focus on the mechanism of anoikis in the occurrence and metastasis of cancer, but the relationship between ARGs and cancer prognosis remains to be studied.

In our study, we found some unique ARGs based on TCGA database to predict the survival prognosis of SKCM patients. By using LASSO-Cox regression, a predictive signature was established based on the training cohort, and the robustness of this model was verified in the testing cohorts. The model consisted of 6 ARGs, including BMP6, CRABP2, FASLG, KRT14, NOX4, and SATB1. Cellular retinoic acid-binding protein 2 (CRABP2) transports retinoic acid from cytoplasm to nucleus, and interacts with nuclear receptors to regulate gene expression Increased levels of CRABP2 have been found in various types of cancer, and are associated with poor survival prognosis.^[27]^ A recent study found that CRABP2 is overexpressed in thyroid malignant tumors, and promotes the invasiveness of thyroid cancer and the anoikis resistance via the integrin/FAK/AKT pathway.^[28]^ FASLG is a type II transmembrane protein, with its C-terminal extracellular domain responsible for interacting with its cognate receptor FAS, and its polymorphism was considered to be related to cancer risk.^[29]^ High expression of KRT14 (keratin 14) in human lung cancer was associated with nodal metastasis and poor survival. K14-high cells contribute to lung cancer metastasis potentially through inhibition of anoikis via upregulation of Gkn1.^[30]^ The upregulation of NADPH oxidase 4 (NOX4) may promote anoikis resistance via ROS generation and the activation of EGFR.^[31,32]^ Wang et al^[33]^ demonstrated that the expression of SATB1 and anoikis resistance could suppress the growth and metastasis of human hepatocellular carcinoma, which was induced by hepatitis B virus (HBV)-encoded viral protein HBx. In summary, an enormous number of studies have shown that the model genes are closely related to anoikis, providing theoretical and compelling support for our risk model.

Furthermore, samples in all sets were divided into high-risk and low-risk groups in accordance with the median risk score, and univariate and multivariate Cox regression analyses were applied to prove our signature was an excellent predictive indicator for estimating the OS, PFS, and DSS of SKCM patients. Subsequently, in order to detect the relationship between the prognostic model and other clinical features, a nomogram was established. The nomogram combines the risk score with other independent clinical factors, sets up an individualized scale, and more comprehensively quantifies and visualizes the risk of different individuals.

Current studies have shown that malignant melanoma is highly sensitive to the immune regulation of the body. The susceptibility of its immune system activation was mainly due to its high tumor mutation load and cancer testis caused by ultraviolet radiation, as well as the expression of antigens and simulation of melanocyte lineage proteins and pathogen-associated antigens.^[34,35]^ At present, immunotherapy has become one of the most important treatments for patients with advanced SKCM. Since melanoma is a highly aggressive tumor that has natural characteristics of immune escape,^[1]^ identifying biomarkers new within the TME may formulate the effective cancer treatment strategies. In this study, we analyzed the differences in the TME and responsiveness to immunotherapy between high-risk and low-risk groups, which has a certain significance for guiding individualized immunotherapy of SKCM.

Finally, although our prediction model shows favorable prediction efficiency, it still has certain limitations. As a retrospective study, the number of samples and obtained clinical data are limited, so the selection of variables is still limited. A series of experiments are needed to evaluate the prognostic value of the 6 ARG signature more convincingly.

## 5. Conclusion

In this study, we established a novel signature in predicting prognosis and immunotherapy with satisfactory results. It can be considered reliable and to formulate the complex treatment for SKCM patients.

## Author contributions

**Conceptualization:** Ziqian Xu.

**Data curation:** Ziqian Xu, Juntao Huang.

**Formal analysis:** Ziqian Xu.

**Investigation:** Ziqian Xu, Juntao Huang, Weimin Shi, Bingjiang Lin.

**Methodology:** Ziqian Xu, Juntao Huang, Weimin Shi, Bingjiang Lin.

**Writing—original draft:** Ziqian Xu, Juntao Huang.

**Software:** Juntao Huang, Feng Yuan.

**Supervision:** Juntao Huang, Feng Yuan.

**Validation:** Juntao Huang, Ying Qi, Feng Yuan.

**Writing—review & editing:** Juntao Huang.

**Project administration:** Weimin Shi, Bingjiang Lin.

**Resources:** Weimin Shi, Bingjiang Lin.

**Visualization:** Ying Qi, Feng Yuan.

## Supplementary Material


