# Supplementary material for: Identification of an anoikis-related gene signature and characterization of immune infiltration in skin cutaneous melanoma
Source: Medicine (Baltimore). 2024 Apr 26;103(17):e37900. doi: 10.1097/MD.0000000000037900 (PMC11049774; doi:10.1097/MD.0000000000037900)
Supplement: Supplementary file 1 [file medi-103-e37900-s001.doc]

**Supplementary Table 1 Anoikis-related genes from the GeneCards database.**

BRMS1 PTK2 NTRK2 BCL2L11 SRC CEACAM6 CAV1 AKT1 ITGB1 CEACAM5 EGFR BCL2 CASP8 SIK1 PTRH2 STAT3 TLE1 DAPK2 CTNNB1 ZNF304 MAPK1 BMF ITGA5 TP53 MCL1 BCL2L1 CASP3 CDH1 BAD PIK3CA PAK1 ITGAV FN1 MAPK3 PTGS2 BAX BCAR1 PTEN ERBB2 PDK4 ANGPTL4 CYCS BRAF YAP1 ANKRD13C ITGA2 ANXA5 BIRC5 MTOR TIMP1 BDNF CSPG4 BSG AKT2 STK11 IGF1 IGF1R ITGA6 ILK CFLAR RHOA HIF1A DAP3 MYBBP1A ITGA3 TLE5 PTK2B CCND1 CTTN CALR ATF4 CDCP1 SKP2 CHEK2 HGF E2F1 EGF PIK3CG ITGB4 DAPK1 PIK3R1 PIK3R3 MAP2K1 CXCL12 LGALS3 FBXW7-AS1 BAK1 ABHD4 CD44 ITGA4 FADD PHLDA2 TGFB1 HMCN1 MMP2 CEBPB CEMIP CDKN3 CBL CASP9 SFN MTDH PRKCA TNFRSF10B CXCL8 MIR200C AR CDKN2A MAPK8 CPT1A PIK3CB CLDN1 MIR204 MIR26A1 CDKN1A CDKN1B KLF12 NTRK1 PLAU MYC PLK1 SMAD4 MUC1 PLAUR LGALS1 PYCARD SESN2 ITGB3 KRAS THBS1 BID HRAS CDK11B CDK11A XIAP PPARG IL6 MIR145 CCR7 MSLN RAC1 GRHL2 NOTCH1 RHOG CCAR2 NQO1 BIRC3 MMP13 FAS MTA1 MYO5A EDA2R CCN6 MMP9 ABL1 MAPK11 PTHLH PDGFB GLI2 EZH2 CXCR4 RIPK1 HMGA1 SIK2 TNFSF10 ANGPTL2 S100A4 ETV4 NTF3 MIR21 MIR124-1 HTRA1 LATS1 CEACAM3 EIF2AK3 LAMC2 LAMA3 LAMB3 CDH2 CSNK2A1 EDIL3 ZEB2 TLN1 EPHA2 SOD2 SIRT3 OLFM3 CLU SPINK1 CPEB2 NAT1 TSG101 MIR200A MIR6744 SERPINA1 AKT3 RELA TNFRSF1A AFP FASLG EEF1A1 ITGA8 SATB1 CD63 LTB4R2 NOX4 PBK MAVS HRC RHOB CCN2 PPP1R13B PLG MET RAF1 PARP1 PRKCQ BRCA2 RB1 DOCK1 HAVCR2 SP1 VTN INHBB PDCD4 PRPF4B RANBP9 SESN1 SESN3 ZBTB7A CD24 MIR141 ELANE KDR MDM2 NFE2L2 PRKCI ZEB1 HK2 KL CRYAB EPHB6 FGF2 LTF IQGAP1 MGAT5 SDCBP ABHD2 SPIB TRIM31 MIR1827 PDGFRB TLR3 PLAT ROCK1 NRAS CASP10 PAK4 VEGFA PIN1 YWHAZ TWIST1 UBE2C IL1RAP BMP6 ELK1 PRDX4 BNIP3 BNIP3L KDM3A LMO3 ZNF32 MIR200B MIR525 MIR363 TUBB3 HSP90B1 PTPN11 SLC2A1 HMOX1 PRKACA PAK3 PIK3R2 PPP2CA CASP6 CD36 CDH3 LRP1 PTK6 EEF2K GLO1 LPAR1 PAK2 ADCY10 RBL2 CEACAM1 GDF2 SIRPA TRAF2 APOBEC3G MNX1 TNFRSF12A VPS37A BAG1 IL17A COL13A1 RAD9A IFI27 MEGF11 ITPRIP BCL2L15 SNAI2 GLUD1 NOTCH3 PTPN1 FASN MYH9 RPS6KB1 SIRT1 TPM1 PPP2R1A COL4A2 CTNND1 MMP11 CD151 PPP2R2A SEMA7A ARHGEF7 BST2 PPP2R5A PPP2R2D CCN1 CCDC178 MIR10A MIR30C1 MIR30B SHC1 BUB1 CDC25C BUB3 FER ITGB5 SETD2 TP73 CDK1 SLCO1B3 BCL2L2 MAD2L1 DLG1 PDCD6IP EDAR SCRIB SH3GLB1 TDGF1 DYNLL2 TSC2 BAG4 MAP3K7 F10 F3 ADAMTSL1 SERPINB1 MIR181A1 MAP3K1 CTBP1 CEACAM4 PXN MALAT1 IKBKG TFDP1 CRYBA1 SERPINE1 FOXO3 ACTG1 ARHGDIA EZR SLC39A6 BIN1 TIAM1 PDPK1 SMAD7 NTRK3 RHOC CASP2 TNC IRF6 HOTAIR GNE XAF1 SFRP1 MAP2K2 CSK PIK3C2B FOXC2 TAGLN ARHGDIB ENDOG FBLIM1 CCDC80 RACK1 PRKD1 LDHA ANXA2 SMARCE1 SPP1 QSOX1 RBFOX2 RPS6KA3 CDC42 MAOA PIP5K1C JUP ATF2 NKX2-1 NDRG1 CRABP2 ID2 OCLN CEACAM8 PITPNC1 AFAP1L1 INSR HSPB1 PCNA GSK3B NGF TP63 CTNNA1 KRT14 SPHK1 EHMT2 OGT RAC3 SIRT6 ACP1 STK38 FOXA1 MUC4 RHOQ ONECUT1 S100A7 GKN1 SRSF3 MIR107 MIR630 LCK CDK2 DNMT1 MERTK UCHL1 BRCA1 MMP3 ACTB NOS2 SLC2A2 USP9X FYN ROR1 HSPA1A HTRA2 SKI SPTA1 PRDM1 TPP2 C5AR1 CENPF LATS2 SNAI1 TJP1 XRCC5 CDX2 CLDN18 DOK2 IKZF3 S100A11 SERPINB5 THY1 USP11 HOXA10 LGALS8 SNCG HTRA3 SLPI SRPX2 IRX1 CXCL14 EFHD2 KIF18A ZG16B SBSN MIR223 MIR99A MIR451A MIR503 MIR7-1 SNORA80E
